# Effectiveness of the Fiocruz recombinant ChadOx1-nCoV19 against variants of SARS-CoV-2 in the Municipality of Botucatu-SP

**DOI:** 10.3389/fpubh.2022.1016402

**Published:** 2022-10-13

**Authors:** Sue Ann Costa Clemens, Carlos Magno Castelo Branco Fortaleza, Madeleine Crowe, Karen Ingrid Tasca, André Gasparini Spadaro, Jayme Augusto Souza-Neto, Rejane Maria Tommasini Grotto, Regina Sider, Jose Jimeno, Thomas Verstraeten, Ralf Clemens

**Affiliations:** ^1^Department of Pediatrics, Oxford University, Oxford, United Kingdom; ^2^Medical School, Institute for Global Health, Siena University, Siena, Italy; ^3^Department of Infectious Diseases, Botucatu Medical School, São Paulo State University (Universidade Estadual Paulista), Botucatu, São Paulo State, Brazil; ^4^P95 Epidemiology & Pharmacovigilance, Leuven, Belgium; ^5^Botucatu Health Department, Botucatu, São Paulo State, Brazil; ^6^Department of Biotechnology, Faculty of Agronomical Sciences, São Paulo State University (Universidade Estadual Paulista), Botucatu, São Paulo State, Brazil; ^7^Department of Clinical Medicine, Botucatu Medical School, São Paulo State University (Universidade Estadual Paulista), Botucatu, São Paulo State, Brazil; ^8^Intrials, São Paulo, Brazil; ^9^Vaxtrials, São Paulo, Brazil; ^10^International Vaccine Institute (IVI), Seoul, South Korea

**Keywords:** COVID-19, vaccines, ChadOx1, effectiveness, “real world” study, variants of concern

## Abstract

**Introduction:**

As the COVID-19 pandemic progresses, rapidly emerging variants of concern raise fears that currently licensed vaccines may have reduced effectiveness against these new strains. In the municipality of Botucatu, São Paulo State, Brazil, a mass vaccination campaign using ChadOx1-nCoV19 was initiated on 16th of May 2021, targeting people 18–60 years old. Two vaccine doses were offered 12 weeks apart, with the second delivered on 8th of August, 2021. This setting offered a unique opportunity to assess the effectiveness of two ChadOx1-nCoV19 doses in a real-life setting.

**Materials and methods:**

Data on testing, hospitalization, symptoms, demographics, and vaccination were obtained from the Hospital das Clínicas da Faculdade de Medicina de Botucatu. A test-negative study design was employed; whereby the odds of being vaccinated among cases vs controls were calculated to estimate vaccine effectiveness (VE; 1-OR). All individuals aged 18–60 who received a PCR test after the 16th of May and were unvaccinated prior to this date were included in the analysis until the study ended in mid-November 2021.

**Results:**

77,683 citizens of Botucatu aged 18–60 received the first dose, and 74,051 received a second ChadOx1-nCoV19 dose 12 weeks later for a vaccination coverage of 84.2 and 80.2%, respectively. Of 7.958 eligible PCR tests, 2.109 were positive and 5.849 negative. The VE against any symptomatic infection was estimated at 39.2%, 21 days after dose 1, and 74.5%, 14 days after dose 2. There were no COVID-19-related hospitalizations or deaths among the 74,051 fully vaccinated individuals. The VE against severe disease was estimated at 70.8 and 100% after doses 1 and 2, respectively. 90.5% of all lineages sequenced between doses 1 and 2 (16th of May−7th of August) were of the Gamma variant, while 83.0% were of the Delta variant during the second period after dose 2 (8th of August−18th of November).

**Discussion:**

This observational study found the effectiveness of ChadOx1-nCoV19 to be 74.5% against COVID-19 disease of any severity, comparable to the efficacy observed in clinical trials (81.3% after dose 2), despite the dominance of the Gamma and Delta VoCs. No COVID-19-related hospitalizations or deaths in fully vaccinated individuals were reported.

## Introduction

In a recent study, the World Health Organization (WHO) estimated that between 13.3 and 16.6 million people worldwide had died in 2020–2021 because of the COVID-19 pandemic, more than 2.5 times the number of reported COVID-19 deaths (1). The excess death rate ranged from as high as 1.031/100 k population in Eastern Europe, to no excess in some smaller countries in various geographies. The excess rate in Brazil is estimated to be 357/100 k—similar to countries like the US or Italy (2). Large scale vaccination was core for containing the pandemic, including deaths and hospitalizations. The most used COVID-19 vaccine globally is the vector construct ChadOx1-C0V19 developed by the University of Oxford.

As the pandemic progresses, variants of concern (VoCs) of SARS-CoV-2 are constantly emerging; and mutations promoting immune escape have raised questions about the effectiveness of currently licensed COVID-19 vaccines against these emerging VoCs. In January 2021, the P.1 variant (also known as the Gamma variant), designated a VoC by the WHO due to its increased transmissibility, was first identified in Japan in travelers arriving from Manaus, Brazil (3). By May 2021, the Gamma variant was the dominant strain circulating in Brazil.

In early 2021 one vaccine against COVID-19—ChadOx1-nCoV19—was licensed in Brazil and the inactivated COVID-19 vaccine Coronavac was approved for emergency use in Brazil. Both vaccines have shown clinical efficacy: Coronavac showed an overall efficacy of 50.7% against symptomatic disease of any severity after two doses (4), while ChadOx1-nCoV19 demonstrated an efficacy of 81.3% against symptomatic infection after two doses given more than 12 weeks apart and of 76.0% 3 weeks after single dose vaccination (5).

At the time of the predominance of Gamma variant circulation in Brazil, there was little evidence of the clinical efficacy or effectiveness of COVID-19 vaccines against this variant (6). In two preclinical studies, sera from ChadOx1-nCoV19 vaccines demonstrated a similar neutralization capacity against the Gamma variant as for Alpha (also known as B.1.17), whereas sera from Coronavac vaccines showed a complete absence of neutralization activity against the Gamma variant (7).

Mass vaccination campaigns offer a unique opportunity to conduct observational studies on the effectiveness and safety of the intervention. With the support from the Brazilian Ministry of Health (MoH), we conducted a test-negative design (TND) observational study on the effectiveness of the recombinant COVID-19 vaccine ChadOx1-nCoV19 from Oxford/Fiocruz as part of a large-scale vaccination campaign in Botucatu, Brazil. Botucatu is a city in inner São Paulo State with a population of 1,42,092 inhabitants, according to the São Paulo Demographic and Statistical Foundation (www.seade.gov.br). The city harbors a university hospital that provides tertiary care for surrounding municipalities, an area comprising half a million people. The municipal health department adheres to the family health program within Brazil's socialized Unified Health System (SUS); and historically has had high adherence to previous vaccination campaigns.

This setting thus offered a unique opportunity to assess the overall effectiveness of 1 and 2 doses of ChadOx1-nCoV19, and specifically against new variants, in a real-life setting.

## Materials and methods

### Study population and design

The Brazilian MoH launched a mass vaccination campaign using ChadOx1-nCoV19 starting on the 16th of May 2021, in the municipality of Botucatu, Brazil, targeting the population between 18 and 60 years of age. At the time of the study, vaccination in the 18–60-years-old population was not yet implemented in Brazil, except for health care workers (HCWs) and high risk citizens. All citizens in this age group (*N* = 92,349) were eligible for inclusion in the campaign if not yet vaccinated, including those with underlying conditions. Vaccination was offered by qualified HCWs in four school courts and, importantly, in 45 election-voting locations with which the vaccinees were familiar, to facilitate campaign adherence. A second dose was planned to be administered 12 weeks after the first dose. Whilst there was no lockdown during the entire study period, mask use was mandatory throughout the study period.

### Objectives and outcomes

The main objective of the study was to determine the vaccine effectiveness (VE) of ChadOx1-nCoV19 against any COVID-19 disease, by dose, and VoC. The VE against severe COVID-19 disease (defined as hospitalization or death) was a secondary objective.

All individuals fitting the clinical criteria for COVID-19 as established by the municipal secretary of health according to globally accepted standards received one of three types of tests: a PCR test, a rapid antigen test, or a serology test. PCR positive samples were sequenced upon consent from the participant. All participants who received a positive or negative PCR test result after the 16th of May, 2021, and were unvaccinated prior to the same date, were included in the analysis.

### Study design

In order to measure VE, a TND was applied, whereby the odds of being vaccinated were compared among cases (testing positive for SARS-CoV-2) and controls (not testing positive). Logistic regression was used to obtain the odds ratio and the VE (1−*OR*). The VE was calculated from 21 days after dose 1 and 14 days after dose 2, as efficacy has been reported to occur after this interval (5).

### Data sources

Records containing information on COVID-19 testing and vaccination status were entered into case report forms (CRFs). Individual information was derived from four de-identified databases for the period from the 17th of May to the 18th of November 2021: (1) E-SUS, used for routine influenza-like-illness (ILI) surveillance, adapted for the COVID-19 pandemic and containing information on symptomatic and asymptomatic cases, (2) SIVEP, containing information on severe, hospitalized ILI cases; (3) VACIVIDA, containing vaccination information on all vaccinated individuals, and (4) a database containing information on testing and sequencing results. Databases were linked using a randomly generated de-identified ID.

### Statistical analysis

The analysis was restricted to those that received an RT-PCR test within 10 days of symptom onset, to account for the reduced sensitivity of the PCR test after this time (8). Analysis was based on illness episodes. Multiple tests taken on 1 day were counted as one test, with the positive result counting toward the analysis if one was present. Any tests taken within 21 days of each other were counted as one illness episode. Individuals who received more than two vaccine doses were excluded from the analysis. Individuals who received a positive test result prior to being vaccinated were counted as unvaccinated. We adjusted for sex, week of symptom onset, age group (by 10-year brackets, i.e., 18–30 years, 31–40 years, 41–50 years, and 51–60 years), and comorbidities as per protocol. As a sensitivity analysis, VE was also estimated from an intent-to-treat (ITT) perspective, measured from the first day after each dose.

### Ethics approval

The research was approved by the Ethical Committee of Botucatu Medical School/São Paulo State University (FMB/UNESP) and by the Comissao Nacional De Etica em Pesquisa CONEP. Only participants who consented to having their information collected were enrolled in the study. During the statistical analysis phases, only de-identified data were received for analysis.

## Results

A total of 65,450 citizens aged 18–60 years received a first dose of ChadOx1-nCoV19 on the first day of the campaign (Sunday, 16th of May), and 12,233 in the following 4 weeks, for a total of 77,683 vaccines (84.2% coverage). Of these, 60,333 citizens received their second ChadOx1-nCoV19 dose 12 weeks later, on Sunday, 8th of August, with an additional 13,718 citizens over the next 4 weeks, for a total of 74,051 fully vaccinated persons (80.2% coverage).

**Figure 1:**
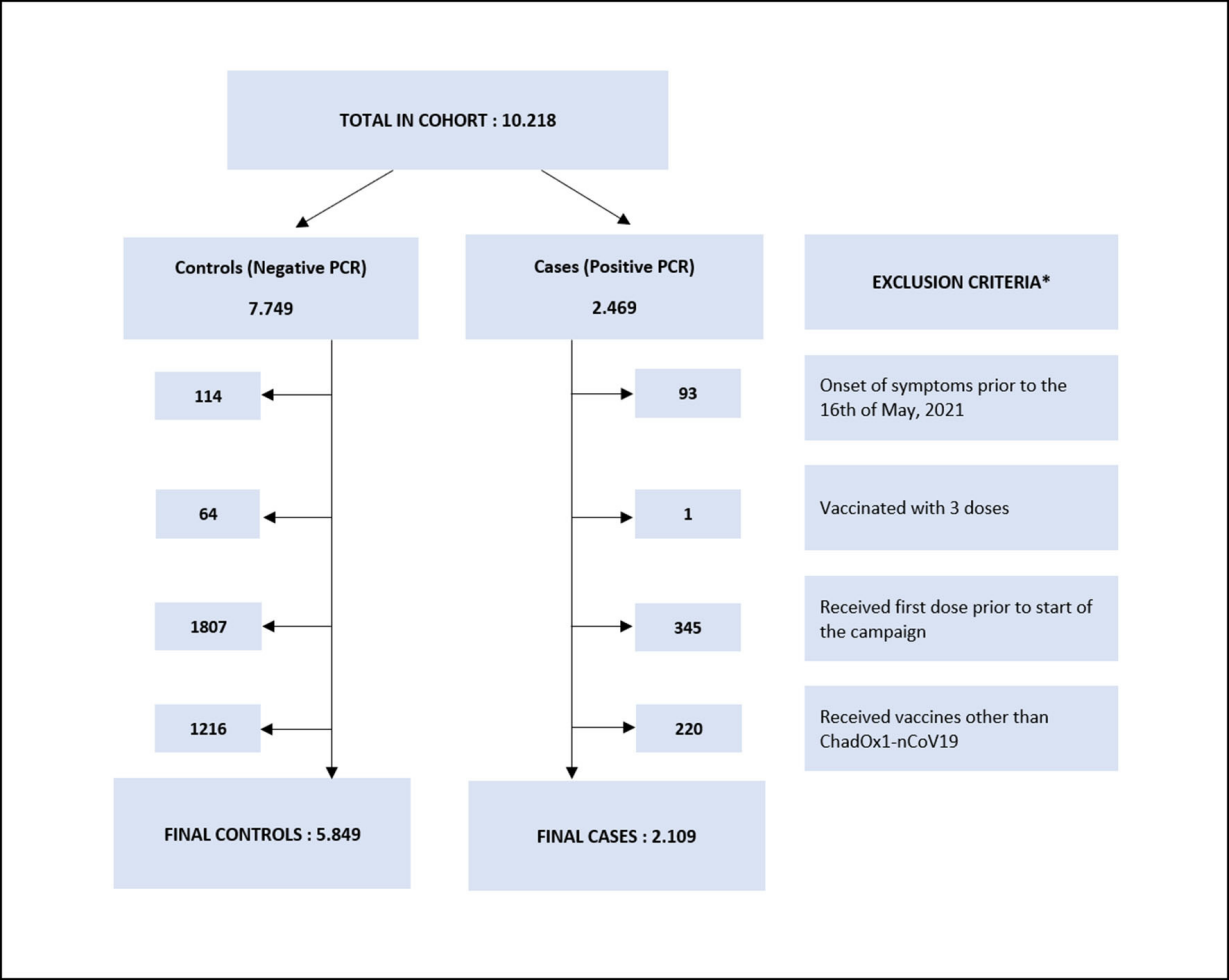
Exclusion criteria applied to overall cohort. *Exclusion criteria may overlap.

A total of 10,218 PCR tests were available and successfully linked to vaccination data. After various exclusion criteria were applied, 7,958 PCR results were available for analysis, of which 2,109 positive PCRs were recorded in people 18–60 years old (Figure 1).

Table 1 shows the baseline characteristics of the study population. The mean age was 35.7 years for the controls and 37.9 for the confirmed COVID-19 cases. Gender was approximately equally distributed in both groups. The positivity rate of the PCR was highest (48.2%) in the week immediately following the first campaign, and rapidly declined to below 20% following the second campaign and finally below 10% in October.

**Table 1:** Baseline characteristics of persons 18–60 years old tested for COVID-19.

	**Controls**	**%**	**Cases**	**%**
*N* ^*^	5,849	73.5%	2,109	26.5%
Age mean (sd)	35.7 (11.3)		38.0 (11.5)	
Age median	34.7		37.9	
**Gender**				
Female	3,240	55.4%	1,096	52.0%
**Age group**
18–30 years	2,147	36.7%	640	30.4%
31–40 years	1,618	27.7%	536	25.4%
41–50 years	1,277	21.8%	537	25.5%
51–60 years	807	13.8%	396	18.8%
Calendar week of test^*^				
17–23 May 2021	325	51.8%	303	48.2%
24–30 May 2021	479	58.6%	339	41.4%
31 May−6 June 2021	492	55.2%	400	44.8%
7–13 June 2021	482	65.0%	259	35.0%
14–20 June 2021	386	76.6%	118	23.4%
21–27 June 2021	308	78.6%	84	21.4%
28 June−4 July 2021	246	80.9%	58	19.1%
5–11 July 2021	196	79.0%	52	21.0%
12–18 July 2021	232	85.9%	38	14.1%
19–25 July 2021	198	78.9%	53	21.1%
26 July-1 Aug 2021	167	79.5%	43	20.5%
2–8 Aug 2021	239	77.3%	70	22.7%
9–15 Aug 2021	231	78.8%	62	21.2%
16–22 Aug 2021	197	82.8%	41	17.2%
23–29 Aug 2021	175	82.9%	36	17.1%
30 Aug−5 Sep 2021	172	87.8%	24	12.2%
6–12 Sep 2021	114	75.0%	38	25.0%
13–19 Sep 2021	172	85.1%	30	14.9%
20–26 Sep 2021	191	86.4%	30	13.6%
27 Sep−3 Oct 2021	157	93.5%	11	6.5%
4–10 Oct 2021	142	96.6%	5	3.4%
11–17 Oct 2021	104	97.2%	3	2.8%
18–24 Oct 2021	123	98.4%	2	1.6%
25–31 Oct 2021	118	98.3%	2	1.7%
1–7 Nov 2021	78	97.5%	2	2.5%
8–14 Nov 2021	94	97.9%	2	2.1%
15–21 Nov 2021	31	88.6%	4	11.4%
**Comorbidities**				
Yes	624	11.0%	235	11.5%

* Percentage in relation to the number tested in the same week.

### Sequencing results

Of the 452 strains with sequencing results, 70.4% were Gamma VoC. The remaining strains were either Delta VoC (27.8%, including B.1.617 or AY variants), or Alpha VoC (1.8%). There was, however, an important shift from Gamma VoC dominance to Delta VoC dominance during the study. Table 2 shows the VoC distribution by time period.

**Table 2:** VoC distribution by time period.

**VoC**	**Period**	
	**Dose 1** **(16th of May−7th of Aug)** ***N* (%)**	**Dose 2** **(8th of Aug−18th of Nov)** ***N* (%)**
Alpha	8 (2.4)	0 (0)
Delta	23 (7.0)	103 (83.0)
Gamma	297 (90.5)	21 (16.9)

### Overall VE

Overall VE against any illness was estimated at 39.2% (95% CI 23.7–51.5) as of 3 weeks after dose 1, and 74.5% (95% CI 23.7–51.5) as of 14 days after dose 2 (Table 3).

**Table 3:** Vaccine effectiveness against any COVID-19 illness, overall and by strain, multivariate analysis only.

**Vaccination status, dose**	**Test-negative status**		**Vaccine effectiveness (95% CI)**		
	**Controls**	**Cases**	**Overall**	**Delta variant**	**Gamma variant**
Unvaccinated	381	185	Reference		
Dose 1	2,171	609	39.2 (23.7–51.5)	35.9 (−45.3 to 70.4)	51.0 (21.6–68.6)
Unvaccinated	70	24	Reference		
Dose 2	1,359	122	74.5 (23.7–51.5)	76.0 (49.5–87.8)	81.3 (−298.0 to 97.9)

A total of 73 patients with PCR-confirmed COVID-19 infections had severe COVID-19 disease: 64 hospitalizations and nine deaths. None of the 74,051 citizens who were fully vaccinated with ChadOx1-nCoV2 was hospitalized or died during the 3 months of observation. Ten hospitalizations and two deaths occurred in one-dose recipients, of which eight occurred within the first 3 weeks after the first dose. The remaining 61 severe disease events occurred all in non-vaccines. The VE against severe COVID-19 disease was thus estimated at 70.8% after dose 1 (95% CI 9.6–90.6%) and 100% (95% CI 44.3–100%) after dose 2.

Vaccine effectiveness against Gamma was estimated at 51.0% (95% CI 21.6–68.6%) following dose 1, and VE against Delta was 76.0 (95% CI 49.5–87.8) following dose 2. We were not able to estimate effectiveness against the Alpha variant, as only a total of eight events occurred. We could also not determine the precise VE against Gamma after two doses nor against Delta after one dose due to the shift in epidemiology with insufficient events and statistical power for those respective periods (Table 2).

The ITT analysis whereby VE was measured as of the day of vaccination showed an overall VE after the first dose of 30.7% (95% CI 18.9–40.8) and after the second dose of 70.2% (95% CI 53.0–80.8).

We had defined severe disease in this observational study as hospitalization or death. In the absence of sufficient hospitalizations or deaths among the much smaller cohort of control, we could not estimate with reasonable precision the VE against severe disease. A total of 73 patients with PCR-confirmed COVID-19 infections had severe COVID-19 disease: 64 hospitalizations and nine deaths. None of the 74,051 citizens who were fully vaccinated with ChadOx1-nCoV2 was hospitalized or died during the 3 months of observation. Two deaths and 10 hospitalizations occurred in one-dose recipients, all other severe disease events in non-vaccinees.

## Discussion

This study aimed to estimate the effectiveness of ChadOx1-nCoV19 following a mass vaccination campaign in Botucatu, Brazil, during which 84.2% of the eligible population of 18–60-year-old citizens received at least one dose and 80.2% received a second dose 3 months later. The vast majority of the latter received their first and second dose on single Sundays in May and August in a set-up where the 45 voting stations for elections of the city were transformed into vaccination centers. The vaccine showed an overall effectiveness of 39.2% from week 3 after dose 1, which increased to 74.5% 2 weeks after dose 2, against any COVID-19 illness in individuals 18–60 years old.

This study adds to the body of evidence of VE during active circulation of emerging VoCs, such as Gamma and Delta which were predominant during the study period. In randomized control trials, the clinical efficacy of ChadOx1-nCoV19 showed a 76.0% reduction in symptomatic illness in the 3 weeks after dose 1, and of 81.3% 14 days after dose 2 (9). This, however, occurred in a controlled setting, and with the ancestor strain Wuhan as the predominant strain, prior to the emergence of the Gamma and Delta VoCs.

The effectiveness of ChadOx1-nCoV19 against variants, including Delta, has been reported elsewhere. Sritipsukho et al. (10) reported a real-life VE against Delta to be <50% after dose 1 and 83% after dose 2 of ChadOx1-nCoV19. Besides, a recent systematic review and meta-analysis described that a non-replicating vector vaccine could achieve a VE of 65% against Delta and 63% against Gamma VoCs (11). However, studies relating to the effectiveness of ChadOx1-nCoV19 against Gamma in real-life settings remain sparse. We were therefore specifically interested in estimating VE against Gamma. The VE against Gamma following dose 1 was estimated at 51.0%. We were not able to precisely estimate the VE against this VoC following dose 2 given the epidemiological shift to Delta in that observation period. Hitchings et al. estimated a VE of 33.4% against symptomatic infection 28 days after the first dose of ChadOx1-nCoV19, and of 77.9% 14 days after the second dose, in adults aged over 60 years of age. This study was conducted in the context of Gamma circulation in São Paulo, Brazil; however, no individual sequencing data were available for analysis (6). We found slightly higher VE estimates against any COVID-19 disease and Gamma following the first dose, with slightly longer follow-up time.

Bernal et al. (12) noted a reduced effectiveness against Delta when compared with the Alpha strain with an estimated effectiveness of ChadOx1-nCoV19 for Delta after dose 1 at 30.0% (vs 48.7% for Alpha) and 67.0% (vs 74.5% for Alpha) after dose 2. Our results suggest a slightly higher VE of 76.0% against Delta, comparable to the originally estimated VE against the Alpha strain.

We also calculated VE from the ITT perspective, whereby VE was estimated from the date of vaccination, which produced slightly lower results. VE was not estimated against severe COVID-19 using the TND case control approach as the recorded number of patients within the respective observation periods was very limited. However, none of the 74,051 citizens who had received a full priming was hospitalized or died.

We restricted the analysis to those that had symptom onset 2 weeks after each of the mass vaccination campaign dates, to avoid that an increase in testing following COVID-like side effects of the vaccine may bias the VE estimates. This finding is supported by an observed increase in Emergency Room attendance and hospitalizations in the 1 week following each campaign, which was noted in our safety results reported elsewhere (13).

Our study is subject to some limitations, both inherent to the study setting, and to the study design. The high vaccination coverage achieved in Botucatu (estimated at 84.2% and 80.2% for the first and second doses, respectively, for the population between 18 and 60 years old) may bias the estimation of VE to the null, as the unvaccinated may also have been protected through herd immunity.

Our analyses were limited to a follow-up period of 14 weeks (104 days) after dose 2. Studies of other COVID-19 vaccines have shown significant waning of VE after about 7 months after the second dose (14–16). On the other hand, estimates of effectiveness have also been shown to be positively correlated with time since vaccination up until a certain point (10). Although it is evident that despite antibody waning, the efficacy of most COVID-19 vaccines against severe disease caused by the Delta VoC is largely maintained, a decline in efficacy is recently noticed against infection and milder disease (17). This study would have benefited from a longer follow-up period to detect any of these effects.

We attempted to reduce any selection biases; however, as this was an observational study residual bias is still possible. The use of the TND attempted to control for any selection bias in health-seeking behavior among study participants. Only people who received a PCR test within 10 days of symptom onset were included in the analysis; however, misclassification of cases/controls due to low sensitivity/specificity of the PCR test, which may affect one variant more than another, is still possible. Given that 70.3% of all sequenced sequences were of the Gamma variant, this reduces the possibility of the latter.

## Conclusion

Vaccine effectiveness against any COVID-19 disease was estimated at 39.2% in the 3 weeks after dose 1, and 74.5% 2 weeks after dose 2 of ChadOx1-nCoV19 following a mass vaccination campaign in Botucatu, Brazil, with no hospitalizations or deaths in fully vaccinated individuals. These results suggest that the ChadOx1-nCoV19 was also highly effective in preventing COVID-19 disease in a real-life setting with various circulating variants, not studied in the original efficacy studies. Combined with the reassuring results on the safety of the same vaccine studied in the same campaign, these effectiveness results show the highly beneficial effect that has been achieved through this mass campaign in Botucatu.

## Data availability statement

The raw data supporting the conclusions of this article will be made available by the authors, without undue reservation.

## Ethics statement

The study was reviewed and approved by Ethical Committee of Botucatu Medical School/São Paulo State University (FMB/UNESP) and by the Comissao Nacional De Etica em Pesquisa CONEP. The patients/participants provided their written informed consent to participate in this study.

## Author contributions

SC, CF, and RC conceptualized the study. SC and RC provided supervision. CF, KT, RG, JJ, AS, and JAS curated the data. MC, TV, and RC did the statistical analysis. SC, CF, and AS acquired funding. MC wrote the original draft. SC, RC, RS, CF, and TV wrote, reviewed, and edited. All authors critically reviewed and approved the final version. All authors confirm that they had full access to all the data in the study and accept responsibility to submit for publication.

## Funding

This research was funded by the Brazilian Council for Scientific and Technological Development (CNPq), grant number 401575/2021-7, and the Bill and Melinda Gates Foundation (BMGF), grant number INV-017149.

## Conflict of interest

Author CF received funding from the Brazilian Council for Scientific and Technological Development. Authors MC and TV were employed by P95 Epidemiology & Pharmacovigilance. Author RS was employed by Intrials. Author JJ was employed by Vaxtrials. The remaining authors declare that the research was conducted in the absence of any commercial or financial relationships that could be construed as a potential conflict of interest.

## Publisher's note

All claims expressed in this article are solely those of the authors and do not necessarily represent those of their affiliated organizations, or those of the publisher, the editors and the reviewers. Any product that may be evaluated in this article, or claim that may be made by its manufacturer, is not guaranteed or endorsed by the publisher.
